# Stereotactic body radiation therapy (SBRT) and respiratory gating in lung cancer: dosimetric and radiobiological considerations

**DOI:** 10.1120/jacmp.v11i1.3133

**Published:** 2010-01-29

**Authors:** Tania De La Fuente Herman, Maria T. Vlachaki, Terence S. Herman, Kerry Hibbitts, Julie A. Stoner, Salahuddin Ahmad

**Affiliations:** ^1^ Department of Radiation Oncology The University of Oklahoma Health Sciences Center Oklahoma City OK USA; ^2^ Department of Radiation Oncology British Columbia Cancer Agency Victoria BC Canada; ^3^ Biostatistics and Epidemiology The University of Oklahoma Health Sciences Center Oklahoma City OK USA

**Keywords:** SBRT, 4D CT, lung cancer, radiobiological modeling

## Abstract

The purpose of this study was to assess the impact of respiratory gating on tumor and normal tissue dosimetry in patients treated with SBRT for early stage non‐small cell lung cancer (NSCLC). Twenty patients with stage I NSCLC were studied. Treatment planning was performed using four‐dimensional computed tomography (4D CT) with free breathing (Plan I), near‐end inhalation (Plan II), and near‐end exhalation (Plan III). The prescription dose was 60 Gy in three fractions. The tumor displacement was most pronounced for lower peripheral lesions (average 7.0 mm, range 4.1–14.3 mm) when compared to upper peripheral (average 2.4 mm, range 1.0–5.1 mm) or central lesions (average 2.9 mm, range 1.0–4.1 mm). In this study, the pencil beam convolution (PBC) algorithm with modified Batho power law for tissue heterogeneity was used for dose calculation. There were no significant differences in tumor and normal tissue dosimetry among the three gated plans. Tumor location however, significantly influenced tumor doses because of the necessity of respecting normal tissue constraints of centrally located structures. For plans I, II and III, average doses to central lesions were lower as compared with peripheral lesions by 4.88 Gy, 8.24 Gy and 6.93 Gy for minimum PTV and 0.98, 1.65 and 0.87 Gy for mean PTV dose, respectively. As a result, the mean single fraction equivalent dose (SFED) values were also lower for central compared to peripheral lesions. In addition, central lesions resulted in higher mean doses for lung, esophagus, and ipsilateral bronchus by 1.24, 1.93 and 7.75 Gy, respectively. These results indicate that the tumor location is the most important determinant of dosimetric optimization of SBRT plans. Respiratory gating proved unhelpful in the planning of these patients with severe COPD.

PACS numbers: 87.55.‐x, 87.55.kd, 87.90.+y

## I. INTRODUCTION

Stereotactic body radiation therapy (SBRT) is a noninvasive alternative to surgery for patients with medically inoperable, early stage non‐small cell lung cancer (NSCLC) with tumor control rates ranging from 80% to 95% at two to three years. It utilizes highly conformal radiation techniques to deliver ablative radiation doses (50 to 60 Gy) in few fractions to tumor while limiting dose to surrounding normal tissues.^(^ ^1^ ^,^ ^2^ ^)^ Although promising short‐term results have been reported, long‐term tumor control and toxicity data are still being compiled.

Radiobiological modeling has been used to predict the biological impact of radiation therapy and to compare treatments utilizing various dose‐fractionation schemes, mostly based on the linear quadratic model (LQM).^(3)^ However, for fraction doses of 5 Gy or greater, actual cell survival curves appear linear instead of down bending as LQM would predict. As a result, the LQM may over estimate cytotoxic effects of SBRT.^(^ ^1^ ^–^ ^4^ ^)^ A universal survival curve (USC) model has recently been proposed, combining the LQM for low‐dose region and the multitarget model asymptote for high‐dose region.^(5)^ Based on the USC concept, the single fraction equivalent dose (SFED) was introduced as the dose delivered in one fraction that would cause the same biological effect as any dose‐fractionation scheme in question.^(5)^

Respiratory motion may significantly influence the accuracy and reproducibility of tumor targeting with radiation. There is increasing evidence that gating respiration results in decreased volumes of irradiated normal lung.^(6)^ This may prove to have significant impact in limiting pulmonary toxicity when few large radiation fractions are used such as in SBRT. 4D CT is one method of assessing respiratory pattern and tumor motion during radiation treatment planning. It allows one to determine tumor motion in three dimensions and, therefore, customize treatment margins for each individual patient. It may also be used to identify the optimal phase of respiratory cycle where the healthy lung may potentially be spared of excessive radiation exposure, minimizing the risk of toxicity. 4D CT may be particularly useful for tumors of the lower lung as their motion may be more pronounced due to their proximity to the diaphragm.^(^ ^7^ ^–^ ^10^ ^)^

The purpose of this study was to quantify the range of motion of lung tumors relatively to their location within the lung parenchyma, and subsequently assess the impact of respiratory gating on tumor and normal tissue dosimetry in patients treated with SBRT.

## II. MATERIALS AND METHODS

### A. Patients

Twenty patients (18 male; 2 female) were treated with SBRT in the Department of Radiation Oncology at our institution. The average group age was 71.5 years (range, 58–85 years). All patients had Stage 1A or 1B NSCLC.^(11)^ Eighteen patients were medically inoperable due to poor pulmonary function, and two because of poor cardiovascular function. Patients were scanned with a GE LightSpeed CT scanner and the Real‐Time Position Management (RPM) Respiratory Gating System ver.1.6.5 (Varian Medical Systems, Palo Alto, CA). The gating technique uses large scan field of view, 120 kV, 400 mA, and 60 sec. The 4D CT scanning images obtained from these 20 patients were used in the present study.

**Figure 1:**
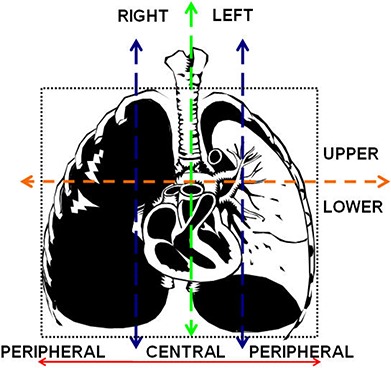
Tumor site and location were classified by dividing the chest cavity vertically along the mediastinum to separate left and right regions, and into three equal portions (peripheral right, central, and peripheral left) at the level of the carina in the coronal orientation. Also, the thorax was segmented by a transversal line at the carina to divide the upper from the lower lung regions. The tumor location seemed central if only tumor margin touched the line defining the central region.

All patients breathed freely during the entire scan and treatment, and no respiratory immobilization was used. The full breathing cycle was divided in ten phases. Plan I was defined by the average of all the ten images acquired during the full breathing cycle. Plan II and Plan III were defined by the average of images obtained during the three segments of respiratory cycle corresponding to near‐end inhalation and near‐end exhalation respectively. The contouring of the tumor and the normal tissues was done separately for each plan. That is, for Plan I, the ITV and normal tissues were contoured on the average image resulting from the entire respiration cycle. For Plans II and III, the ITV and normal tissues were contoured on the average of the images from the near‐end inhalation window and the near‐end exhalation window, respectively. Utilizing the tumor site and location criteria (Fig. 1), 15 patients were classified with peripheral lesions (nine in the upper and six in the lower region) and five patients with centrally located lesions. Tumors were contoured using windows and levels optimized for lung tissue.

### B. Treatment planning

The CT images were sorted using the 4D planning software (GE Advantage Workstation aw4.3_06), and SBRT treatment plans were created using Eclipse Treatment Planning System 7.4 (Varian Medical Systems, Palo Alto CA). The plans for this study used a prescription dose of 60 Gy in three fractions. All plans had five to seven, 6 MV, coplanar non‐opposing beams. The ITVs were given a 0.7 cm circumferential margin to create the PTVs. The standard dose calculation algorithm (pencil beam with modified Batho Power Law) was used. The optimization goals were to deliver 60 Gy to 95% of the PTV, but at least 95% of the prescription dose to 95% of the PTV was allowed to meet all normal tissue constraints, and 99% of the PTV was to receive a minimum of 90% of the dose. Doses 5% above the prescribed dose had to reside within the PTV. Maximum allowed doses to spinal cord, esophagus, heart, and ipsilateral bronchus were 18 Gy, 27 Gy, 30 Gy, and 30 Gy, respectively.^(12)^ For normal lung, the percentage volume receiving 20 Gy or more, $V_{20\text{Gy}}$, was restricted to 10%, unless this proved unworkable, in which case up to 15% was acceptable. The heterogeneity index (HI) was defined as the ratio of the dose at 1% of the PTV to the dose at 99% of the PTV $\left(D_{1\%}/D_{99\%}\right)$.

### C. Radiobiological modeling

Because dose heterogeneities exist within the target volume $\left(D_{1\%}/D_{99\%},\;\text{HI}>1\right)$, the cumulative dose volume histogram (DVH) was used to generate equivalent uniform dose (EUD) and tumor control probability (TCP)^(13)^ assuming a clonogen cell density (CCD)=220 million/cc; ${\text{SF}}^{2}=0.4$, at reference dose=2 Gy; number of fractions =3; Dose=60 Gy; and α/β=10. The calculation of EUD was done with the Niemerko method^(14)^ when fractionation effects using the LQM are also included.^(^ ^14^ ^,^ ^3^ ^)^ The concept of EUD was then incorporated into the calculation of the single fraction equivalent dose (SFED), which seeks to represent the biological effect of any dose‐fractionation scheme of an equivalent single fraction dose.^(5)^

### D. Quantification of tumor displacement

**Figure 2:**
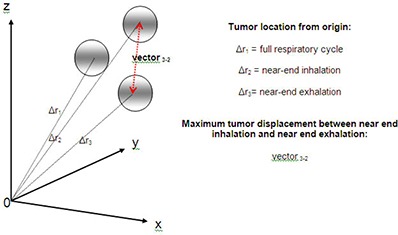
Internal target volume (ITV) location with respect to respiratory gating plans.

To attribute a location to the tumor inside the thorax, the coordinates given by the treatment planning software for the marked CT slice (used for patient setup) were considered the origin ${(x}_{0}{,y}_{0}{,z}_{0})$, and the location of the center of the tumor for Plan I was defined as ${(x}_{1}{,y}_{1}{,z}_{1})$, for Plan II as ${(x}_{2}{,y}_{2}{,z}_{2})$, and for Plan III as ${(x}_{3}{,y}_{3}{,z}_{3})$. The measurement of the vector distance from the origin to the center of each ITV was calculated in the following manner and graphically shown in Fig. 2:

(1) $$\Delta r_{1}=\sqrt{{\left(x_{1}-x_{0}\right)}^{2}+{\left(y_{1}-y_{0}\right)}^{2}+{\left(z_{1}-z_{0}\right)}^{2}}$$

(2) $$\Delta r_{2}=\sqrt{{\left(x_{2}-x_{0}\right)}^{2}+{\left(y_{2}-y_{0}\right)}^{2}+{\left(z_{2}-z_{0}\right)}^{2}}$$

(3) $$\Delta r_{3}=\sqrt{{\left(x_{3}-x_{0}\right)}^{2}+{\left(y_{3}-y_{0}\right)}^{2}+{\left(z_{3}-z_{0}\right)}^{2}}$$

The maximum displacement of the tumor was calculated as follows:

(4) $$\text{vec}tor_{3-2}=\sqrt{{\left(x_{3}-x_{2}\right)}^{2}+{\left(y_{3}-y_{2}\right)}^{2}+{\left(z_{3}-z_{2}\right)}^{2}}$$

### E. Statistical analysis

Statistical analysis was performed using the SAS System for Windows, ver.9.1 (SAS Institute Inc., Cary, NC). The data was summarized using the mean ± standard deviation (SD). The mean volume and dose values were compared among the three treatment plans of respiratory phases using a mixed effects analysis of variance (ANOVA) modeling approach. The mixed model included a random subject effect to account for correlation among multiple measurements made on each subject. The model also included a fixed measurement phase (with three levels) term. The Tukey method was used to make pairwise comparisons between pairs of the phases if the overall test (comparing mean response among the three treatment plans) was significant. A two‐sided 0.05 alpha level was used for hypothesis testing.

## III. RESULTS

### A. Volumes

The group average ITVs for Plans I, II and III, were 17.99 cm^3^ (range, 1.17–73.60 cm^3^), 18.06 cm^3^ (range, 0.67–69.39 cm^3^), and 17.12 cm^3^ (range, 0.97–66.93 cm^3^), respectively. The group average volumes for PTV, uninvolved lung, esophagus, ipsilateral bronchus, heart, and spinal cord are given in Table 1. There was no significant difference in the mean PTV volume measures among the three plans (p=0.2). The mean volume of the normal lung, however, differed significantly among the three Plans (p=0.006). Based on pairwise comparisons, the mean lung volume for Plans I and III was significantly lower compared to Plan II $(4204.85\pm 1260.75{\text{cm}}^{3}$ and $4258.27\pm 1250.37{\text{cm}}^{3}$ versus $4437.70\pm 1213.55{\text{cm}}^{3}$, respectively, p≤0.04). For unclear reasons, mean heart volumes differed significantly among the three Plans (p=0.05), whereas for ipsilateral bronchus, spinal cord, esophagus and ITV, they did not (p≥0.1, respectively).

**Table 1:** Average volumes.

*Structure of Interest*	*Plan I*	*Average Volume (cm^3^) Plan II*	*Plan III*
ITV	17.99	18.06	17.12
PTV	60.48	60.06	57.80
Uninvolved Lung	4204.85	4437.70	4258.27
Esophagus	37.86	37.67	39.93
Ipsilateral Bronchus	2.09	2.36	2.21
Heart	759.99	710.92	712.87
Spinal Cord	55.01	55.03	55.91

### B. Tumor displacement

The average maximum tumor displacement was 4.2 mm (range 1.0–14.3 mm) for all peripheral and 2.9 mm (range 1.0–5.1 mm) for all central lesions. Therefore, the tumor displacements were minimal in this patient population, which was medically inoperable because of severe COPD, but one patient with cardiovascular disease had a displacement of 14.3 mm in a lower peripheral tumor (Table 2.

**Table 2:** Displacement between inhalation and exhalation (left upper lobe (LUL), left lower lobe (LLL), right upper lobe (RUL), and right lower lobe (RLL).

*Tumor Location*	*Patient*	*Displacement (mm) Tumor Site*	*Inhalation to Exhalation*
Peripheral	1	LUL	2.2
	2	RLL	4.1
	3	LUL	1.4
	4	RLL	14.3
	5	RLL	7.3
	6	RUL	2.0
	8	LLL	4.5
	11	RUL	1.0
	12	LUL	1.4
	13	LUL	5.1
	15	RLL	5.1
	16	RUL	3.5
	17	RUL	1.0
	19	LLL	6.8
	20	LUL	3.7
Central	7	RLL	1.0
	9	RLL	2.2
	10	LUL	4.1
	14	RUL	3.2
	18	RUL	3.7

### C. PTV doses

PTV doses for all plans are shown in Table 3. There were no significant differences in PTV doses among the three gated plans. However, central tumor location was associated with significantly lower average minimum PTV doses compared to peripheral lesions for all plans (p≤0.044). Specifically, average minimum PTV doses for upper peripheral, lower peripheral, and central lesions were 53.13, 56.04 and 49.41 Gy for Plan I, 53.40, 56.36 and 46.34 Gy for Plan II, and 53.31, 57.02 and 47.86 Gy for Plan III, respectively. The average mean PTV doses for upper peripheral, lower peripheral, and central lesions were 62.34, 62.70 and 61.50 Gy for Plan I, 63.05, 62.79 and 61.29 Gy for Plan II, and 62.63, 62.41 and 61.67 Gy for Plan III, respectively. The average heterogeneity indices were 1.14 for upper peripheral (range 1.10–1.21), 1.11 for lower peripheral (range 1.08–1.14) and 1.17 for central lesions (range 1.12–1.26), indicating a relatively large heterogeneity in PTV dose distribution.

**Table 3:** PTV dose for different tumor sites, locations, and respiratory combinations.

*Average PTV Dose (Gy)*												
*Respiratory Gating Plans*	*Minimum*				*Mean*				*Maximum*			
	*Peripheral*		*Central*		*Peripheral*		*Central*		*Peripheral*		*Central*	
	*Upper*	*Lower*	*All*	*All*	*Upper*	*Lower*	*All*	*All*	*Upper*	*Lower*	*All*	*All*
Plan I	53.13	56.04	54.29	49.41	62.34	62.70	62.48	61.50	66.71	65.57	66.26	66.54
Plan II	53.40	56.36	54.58	46.34	63.05	62.79	62.94	61.29	67.28	66.09	66.80	68.32
Plan III	53.31	57.02	54.79	47.86	62.63	62.41	62.54	61.67	66.16	65.03	65.71	66.55

### D. Normal tissue doses

**Figure 3:**
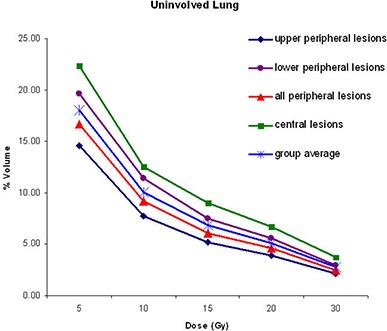
Percentage of uninvolved lung receiving doses of 5, 10, 15, 20, and 30 Gy or higher, obtained from dose volume histograms of plan at full respiratory cycle (Plan I).

The average mean and maximum doses for all the normal tissues according to the tumor location and site are given in Table 4. Central lesions resulted in higher mean doses to the uninvolved lung in all plans (p=0.008). Specifically, average mean lung doses for upper peripheral, lower peripheral, and central lesions were 2.83, 3.85 and 4.47 Gy for Plan I, 2.65, 3.50 and 4.62 Gy for Plan II, and 2.84, 3.59 and 4.56 Gy for Plan III respectively. The average percentage of volume of uninvolved lung receiving doses 20 Gy or higher for upper peripheral, lower peripheral, and central lesions were 3.92%, 5.60% and 6.74% for Plan I, 3.90%, 4.80% and 7.50% for Plan II, and 3.90%, 5.20% and 7.39% for Plan III, respectively (Table 5. The percentages of uninvolved lung volumes receiving doses of 5, 10, 15, 20 and 30 Gy or higher for all lesions with Plan I are graphically presented in Fig. 3.

**Table 4:** Average normal tissue dose for different tumor sites, locations, and respiratory gating plans.

*Normal Tissues*	*Average Mean Dose (Gy)*				*Average Maximum Dose (Gy)*			
	*Upper*	*Peripheral Lower*	*All*	*Central All*	*Upper*	*Peripheral Lower*	*All*	*Central All*
**Plan I**								
Uninvolved Lung	2.83	3.85	3.23	4.47	62.73	62.93	62.81	63.50
Esophagus	1.62	1.83	1.70	3.71	7.81	8.89	8.24	22.20
Ipsilateral Bronchus	3.28	1.42	2.53	10.28	4.70	4.12	4.47	19.91
Heart	0.64	2.18	1.26	0.63	5.93	10.98	7.95	7.56
Spinal Cord	1.02	1.81	1.34	1.81	9.38	10.45	9.81	13.57
**Plan II**
Uninvolved Lung	2.65	3.50	2.99	4.62	62.81	62.77	62.79	64.26
Esophagus	1.48	1.71	1.57	3.71	7.37	8.73	7.92	22.40
Ipsilateral Bronchus	3.35	1.34	2.54	10.66	5.09	3.67	4.52	24.69
Heart	0.67	2.18	1.27	0.51	6.52	10.19	7.99	6.81
Spinal Cord	0.97	1.78	1.30	1.76	8.99	10.48	9.59	13.01
**Plan III**
Uninvolved Lung	2.84	3.59	3.14	4.56	62.15	62.75	62.39	63.36
Esophagus	1.51	1.57	1.53	3.46	8.02	8.44	8.19	22.42
Ipsilateral Bronchus	3.39	1.88	2.79	11.37	6.30	3.92	5.35	25.21
Heart	0.58	2.10	1.19	0.60	4.68	11.57	7.43	6.99
Spinal Cord	1.02	1.68	1.28	1.75	9.36	10.32	9.74	12.97

**Table 5:** Percentage of uninvolved lung covered by 5 Gy, 10 Gy, 15 Gy, 20 Gy, and 30 Gy.

	*Upper Peripheral (%)*	*Lower Peripheral (%)*	*Peripheral (%)*	*Central (%)*
**Plan I**				
V5	14.66	19.66	16.66	22.37
V10	7.73	11.46	9.22	12.57
V15	5.17	7.51	6.11	9.02
V20	3.92	5.60	4.59	6.74
V30	2.18	2.94	2.48	3.74
**Plan II**
V5	13.85	18.15	15.57	22.52
V10	7.34	10.13	8.46	12.99
V15	4.83	6.58	5.53	9.59
V20	3.90	4.80	4.10	7.50
V30	2.01	2.59	2.24	4.14
**Plan III**
V5	14.77	18.26	16.16	22.14
V10	7.86	10.32	8.84	13.13
V15	5.06	6.86	5.78	9.60
V20	3.90	5.20	4.42	7.39
V30	2.22	2.74	2.43	4.06

As expected, for patients with central tumors, the esophagus and the ipsilateral bronchus had higher average mean and maximum doses than those patients with peripheral tumors for all three plans (Table 4. The average mean and maximum doses to the heart were higher for lesions in the lower peripheral region of the lung for all plans. Specifically, average heart doses for upper peripheral, lower peripheral, and central lesions were 0.64, 2.18 and 0.63 Gy for Plan I, 0.67, 2.18 and 0.51 Gy for Plan II, and 0.58, 2.10 and 0.60 Gy for Plan III, respectively. Although doses were higher when tumors were in the lower peripheral region, they were not statistically significant (p=0.8). For all plans, upper peripheral lesions resulted in the lowest average and maximum spinal cord doses.

### E. Radiobiological calculations

The EUD and SFED values were similar among the three gated plans. However, central lesions were associated with lower EUD and SFED when compared to peripheral lesions. Specifically for Plans I, II and III, average EUD values were 57.88, 58.00 and 57.81 Gy for upper peripheral, 59.74, 59.64 and 59.91 Gy for lower peripheral, and 55.51, 53.87 and 54.87 Gy for central lesions, respectively. Similarly, average SFED values for Plans I, II and II were 54.28, 54.40 and 54.21 Gy for upper peripheral, 56.14, 56.09 and 56.31 Gy for lower peripheral, and 51.91, 50.27 and 51.27 Gy for central lesions, respectively. As expected, TCP was 100% in all plans with this ablative dose.

## IV. DISCUSSION

In this study, average tumor motion was slightly more pronounced for lower peripheral. This respiration‐induced tumor motion is more limited in our study compared to other reports where tumor displacement up to 3 cm has been described for lower lung tumors.^(15)^ Most of our patients (90%) had chronic obstructive pulmonary disease which usually results in hyperinflated lungs and, therefore, reduced respiratory motion. The one patient who exhibited greater tumor motion was medically inoperable due to cardiovascular rather than pulmonary disease. Consequently, tumor and normal tissue doses did not differ significantly among the three gated plans.

In this study, the average volume for lung was slightly larger in Plan II. This is attributed to the expansion of the lung and thoracic cavity at near end inhalation. The average volume of the esophagus was slightly smaller in Plan II probably as a result of organ compression from the expanded pulmonary parenchyma at near‐end inhalation. In the case of the heart, it was unknown at what instance (systole or diastole) the image was acquired with respect to inhalation or exhalation. Although the average volume among all three plans differed significantly (p=0.05), based on pairwise comparison, the average volumes under Plans II and III were lower than those of plan I but not significantly (p=0.08 and p=0.09, respectively). The average volume of spinal cord and ipsilateral bronchus were not expected to be different but the variations were probably due to random nature of independent contouring of each plan.

Tumor location, however, influenced tumor and normal tissue dosimetry in this study. Central lesions had lower average minimum PTV doses when compared to peripheral tumors. This was due to the strict enforcement of normal tissue dose limits, especially, because the esophagus, ipsilateral bronchus, and spinal cord are organs with serially structured functional subunits and significant damage in even a relative small segment of the structure would be expected to cause severe organ dysfunction. In a phase II study of SBRT for medically inoperable early stage lung cancer patients treated with 60 to 66 Gy in three fractions, local tumor control at three years was 88.1% with nodal recurrences occurring in 8.6% of patients.^(16)^ Specifically, in this same study, high grade toxicity developed in 27.3% of patients with central tumors versus 10.4% of patients with peripheral lesions. These toxicities included cases of pneumonia, pleural effusion, worsening of pulmonary function tests, hemoptysis, and apnea. In a preliminary report^(17)^ from the above study, a grade three to five toxicity was found more pronounced with central lesions, and this resulted in the exclusion of patients in RTOG 0236 with PTV volumes encroaching on a 2 cm margin around the mediastinum and major airways.

Various investigators have studied the dosimetric and spatial effects on radiation pneumonitis in patients with NSCLC.^(^ ^7^ ^–^ ^10^ ^)^ Dosimetric parameters potentially related to radiation pneumonitis include mean lung dose, $V_{7},V_{10}$ and tumor location. A study of 64 NSCLC patients treated with SBRT at single dose of 20 to 30 Gy^(7)^ demonstrated that the lower lung has increased radiosensitivity and therefore a lower dose threshold for toxicity events compared to upper lung.^(7)^ In another clinical study, mean lung doses ranged from 4 to 7 Gy and $V_{13},V_{20}$, and $V_{30}$ were under 10%.^(18)^ In our study, the lowest mean lung doses were observed in upper peripheral lesions (≤2.84 Gy) and the mean doses for lower peripheral and central lesions were ≤3.85 and ≤4.62 Gy, respectively while $V_{15},V_{20}$, and $V_{30}$ were below 9.6% in all three plans, irrespective of tumor location.

Song et al.^(19)^ reported two cases of bronchial stenosis followed by atelectasis in patients with perihilar tumors treated with SBRT. Another SBRT study by Onimaru et al.^(20)^ reported one patient who developed an esophageal ulcer and died from bleeding. The patient had received 48 Gy in eight fractions; the mean esophageal dose was 10.6 Gy and the radiation doses to 10, 5 and 1 cc of the esophagus were 14.8, 29.9 and 42.5 Gy. In our study, central lesions resulted in higher esophageal doses compared to peripheral lesions in all plans. Specifically, mean esophageal doses ranged from 3.46 to 3.71 Gy for central and from 1.48 to 1.83 Gy for peripheral lesions. The radiation doses to the ipsilateral bronchus contoured from the carina to where it begins to subdivide into the bronchi were higher with central lesions compared to peripheral lesions.

In 2008, Park et al.^(5)^ proposed the concept of USC and SFED for comparison among different dose‐fractionation schemes for SBRT. From our heterogeneous target dose distributions, we calculated EUD^(14)^ and incorporated it into the calculation of SFED using USC approach. In this study, SFED values were lower for central lesions (50.27–51.91 Gy) when compared to peripheral lesions (54.21–56.31 Gy). The biological equivalencies of the SFED values in terms of CFRT delivered in 2 Gy per fraction are 106 and 110 Gy for upper and lower peripheral tumors for all plans, respectively, and 101, 98, and 100 Gy for central tumors in Plans I, II, and III, respectively.

One of the limitations of this study has been the use of pencil beam convolution algorithm with modified Batho Power Law (PBC/modified Batho) for dose calculations with heterogeneity corrections instead of the anisotropic analytic algorithm (AAA), a scatter‐based dose model which achieves increased accuracy in scattered dose calculation. A recent Japanese study^(21)^ reported a discrepancy in dose of about 2% calculated by PBC/modified Batho compared to that calculated by AAA in stereotactic lung irradiation. A prescription dose reduction from 20 Gy per fraction to 18 Gy per fraction in three fractions is now suggested by the quality assurance working group of the phase III ROSEL study^(22)^ when utilizing AAA or collapsed cone convolution (CCC) dose calculation models instead of PBC/modified Batho.

The PBC algorithm was also used in some institutions within a Japanese SBRT study JCOG 0403.^(23)^ Specifically, Teiji Nishio et al. utilized a lung phantom to study the differences between measured doses with film and calculated doses with and without heterogeneity corrections. Without heterogeneity corrections, the planned doses were lower by 10%–18%. The investigators chose calculation algorithm with heterogeneity correction for the JCOG 0403 study due to the availability of this algorithm in all participating institutions. This is the case in our study also, where PBC was the only available calculation algorithm at our institution. In contrast to the Japanese study however, the main focus of our work was to evaluate the impact of three different respiratory gating techniques on tumor and normal tissue dosimetry. Finally, the authors acknowledge that although only five to seven coplanar non‐opposing beams were used in this study for treatment planning, other clinics prefer to apply higher number of beams (coplanar and or non‐coplanar) to further reduce normal tissue dose.

Advancements in biomedical imaging may soon prove to be useful in radiotherapy treatment planning for more accurate definition of tumor and normal tissue volumes. Positron emission tomography (PET) detects tumor metabolic activity and is increasingly used in radiotherapy treatment planning and in evaluating treatment responses.^(24)^ Additionally, there is interest in utilizing single photon emission tomography (SPECT) to identify and protect highly functional pulmonary tissue from high radiation dose exposure.^(25)^ The integration of such technologies with respiratory gating has the potential of improving tumor control while minimizing toxicity for lung cancer patients.

## V. CONCLUSIONS

Tumor location was the most important factor influencing tumor and normal tissue dosimetry in this study of thoracic SBRT in medically inoperable patients with lung cancer. While peripheral lesions may display more displacement with breathing, central lesions are associated with lower tumor doses and higher normal tissue doses due to their proximity to dose‐limiting healthy tissues. There was no significant difference in tumor and normal tissue dosimetry observed among the three respiratory gating plans in this study. This was undoubtedly true because tumor motion was minimal in these medically inoperable patients, predominantly due to severe COPD. Additional studies utilizing more accurate dose calculation algorithms such as AAA are warranted. Although requiring further clinical validation, radiobiological modeling with USC and SFED may prove to be a practical and convenient method for comparing different dose fractionation schemes.

## References

[1] Timmerman R , McGarry R , Yiannoutsos C , et al. Excessive toxicity when treating central tumors in a phase II study of stereotactic body radiation therapy for medically inoperable early‐stage lung cancer. J Clin Oncol. 2006;24(30):4833–39. 17050868 10.1200/JCO.2006.07.5937

[2] Timmerman RD , Kavanagh BD , Cho LC , Papiez L , Xing L . Stereotactic body radiation therapy in multiple organ sites. J Clin Oncol. 2007;25(8):947–52. 17350943 10.1200/JCO.2006.09.7469

[3] Fowler JF . The linear‐quadratic formula and progress in fractionated radiotherapy. Br J Radiol. 1989;62(740):679–94. 2670032 10.1259/0007-1285-62-740-679

[4] Hall EJ . Radiobiology for the Radiologist. 5th ed. Philadelphia (PA): Lippincott Williams & Wilkins; 2000.

[5] Park C , Papiez L , Zhang S , Story M , Timmerman RD . Universal survival curve and single fraction equivalent dose: useful tools in understanding potency of ablative radiotherapy. Int J Radiat Oncol Biol Phys. 2008;70(3):847–52. 18262098 10.1016/j.ijrobp.2007.10.059

[6] Vlachaki M , Castellon I , Leite C , Perkins T , Ahmad S . Impact of respiratory gating using 4‐dimensional computed tomography on the dosimetry of tumor and normal tissue in patients with thoracic malignancies. Am J Clin Oncol. 2009;32(3):262–68. 19433968 10.1097/COC.0b013e318184b33a

[7] Kyas I , Hof H , Debus J , Schlegal W , Karger CP . Prediction of radiation induced changes in the lung after stereotactic body radiation therapy of non‐small‐cell lung cancer. Int J Radiat Oncol Biol Phys. 2007;67(3):768–74. 17097829 10.1016/j.ijrobp.2006.08.066

[8] Hope AJ , Lindsay E , El Naqa I , et al. Modeling radiation pneumonitis risk with clinical, dosimetric, and spatial parameters. Int J Radiat Oncol Biol Phys. 2006;65(1):112–24. 16618575 10.1016/j.ijrobp.2005.11.046

[9] Yorke ED , Jackson A , Rosenzweig KE , et al. Dose‐volume factors contributing to the incidence of radiation pneumonitis in non‐small‐cell lung cancer patients treated with three‐dimensional conformal radiation therapy. Int J Radiat Oncol Biol Phys. 2002;54(2):329–39. 12243805 10.1016/s0360-3016(02)02929-2

[10] Seppenwoolde Y , De Jaeger K , Boersma L , Belderbas JS , Lebesque JV . Regional differences in lung radiosensitivity after radiotherapy for non‐small‐cell lung cancer. Int J Radiat Oncol Biol Phys. 2004;60(3):748–58. 15465191 10.1016/j.ijrobp.2004.04.037

[11] The American Society of Clinical Oncology (ASCO) . Staging with illustrations. Alexandria, VA: ASCO; 2007 Accessed 2007 November. Avialable from http://www.cancer.net/patient/Cancer+Types/Lung+Cancer?sectionTitle=Staging%20With%20Illustrations

[12] American College of Radiology . A phase II trial of stereotactic body radiation therapy (SBRT) in the treatment of patients with medically inoperable stage I/II non‐small cell lung cancer. Radiation Therapy Oncology Group, RTOG 0236; 2006. Philadelphia, PA: ACR; 2006.

[13] Webb S . Physics of Conformal Radiotherapy: Advances in Technology. Bristol and Philadelphia (PA): Institute of Physics Publishing; 1997.

[14] Niemerko A . Reporting and analyzing dose distributions: a concept of equivalent uniform dose. Med Phys. 1997;24(1):103–10. 9029544 10.1118/1.598063

[15] Stevens CW , Munden RF , Foster KM , et al. Respiratory‐driven lung tumor motion is independent of tumor size, tumor location, and pulmonary function. Int J Radiat Oncol Biol Phys. 2001;51(1):62–68. 11516852 10.1016/s0360-3016(01)01621-2

[16] Fakiris AJ , McGarry RC , Yiannoutsos CT , et al. Stereotactic body radiation therapy for early‐stage non‐small‐cell carcinoma: four‐year results of a prospective phase II study. Int J Radiat Oncol Biol Phys. 2009;75(3):677–82. 19251380 10.1016/j.ijrobp.2008.11.042

[17] Timmerman R , McGarry R , Yiannoutsos C , et al. Excessive toxicity when treating central tumors in a phase II study of stereotactic body radiation therapy for medically inoperable early‐stage lung cancer. J Clin Oncol. 2006;24(30):4833–39. 17050868 10.1200/JCO.2006.07.5937

[18] Kavanagh BD, Timmerman R, editors. Stereotactic Body Radiation Therapy. Philadelphia (PA): Lippincott Williams and Wilkins; 2005.

[19] Song DY , Benedict SH , Cardinale RM , Chung TD , Chang MD , Schmidt‐Ullrich RK . Stereotactic body radiation therapy of lung tumors: preliminary experience using normal tissue complication probability‐based dose limits. Am J Clin Oncol. 2005;28(6):591–96. 16317270 10.1097/01.coc.0000182428.56184.af

[20] Onimaru R , Shitaro H , Shimizu A , et al. Tolerance of organs at risk in small‐volume, hypofractionated, image‐guided radiotherapy for primary and metastatic lung cancers. Int J Radiat Oncol Biol Phys. 2003;56(1):126–35. 12694831 10.1016/s0360-3016(03)00095-6

[21] Tachibana M , Noguchi Y , Fukunaga J , Hirano N , Yoshidome S , Hirose T . Influence on dose calculation by difference of dose calculation algorithms in stereotactic lung irradiation: comparison of pencil beam convolution (inhomogeneity correction:batho power law) and analytical anisotropic algorithm [article in Japanese]. Nippon Hoshasen Gijutsu Gakkai Zasshi. 2009;65(8):1064–72. 10.6009/jjrt.65.1064 19721315

[22] Hurkmans CW , Cuijpers JP , Lagerwaard FJ , et al. Recommendations for implementing stereotactic radiotherapy in peripheral stage IA non‐small cell lung cancer: report from the quality assurance working party of the randomised phase III ROSEL study. Radiat Oncol. 2009;4:1. 19138400 10.1186/1748-717X-4-1 PMC2631491

[23] Nishio T , Kunieda E , Shirato H , et al. Dosimetric verification in participating institutions in a stereotactic body radiotherapy trial for stage I non‐small cell lung cancer: Japan clinical oncology group Trial (JCOG 0403). Phys Med Biol. 2006;51(21):5409–17. 17047260 10.1088/0031-9155/51/21/002

[24] Henderson MA , Hoopes DJ , Fletcher JW , et al. A Pilot Trial of Serial 18f‐Fluorodeoxyglucose Positron Emission Tomography in Patients with Medically Inoperable Stage I Non‐Small‐Cell Lung Cancer Treated with Hypofractionated Stereotactic Body Radiotherapy. Int J Radiation Oncology Biol Phys. May 25, 2009. Epub ahead of print. 10.1016/j.ijrobp.2009.02.051 PMC2823932 19473777

[25] Shioyama Y , Jang SY , Liu HH . Preserving functional lung using perfusion imaging and intensity‐modulated radiation therapy for advance‐stage non‐small cell lung cancer. Int J Radiation Oncology Biol Phys. 2007;68(5):1349–58. 10.1016/j.ijrobp.2007.02.015 17446001

